# Supplementary material for: Time-series transcriptome analysis identified differentially expressed genes in broiler chicken infected with mixed Eimeria species
Source: Front Genet. 2022 Aug 8;13:886781. doi: 10.3389/fgene.2022.886781 (PMC9393255; doi:10.3389/fgene.2022.886781)
Supplement: Supplementary file 2 [file DataSheet1.ZIP › 7dpi_GO.Gsea.1625106495923/GOBP_LEUKOCYTE_HOMEOSTASIS.html]

Details for gene set GOBP\_LEUKOCYTE\_HOMEOSTASIS[GSEA]

|  || Dataset | TMM\_7dpi\_gct\_format\_7dpi\_gct\_format.Class\_4dpi.cls #PC\_versus\_NC.Class\_4dpi.cls #PC\_versus\_NC\_repos |
| Phenotype | Class\_4dpi.cls#PC\_versus\_NC\_repos |
| Upregulated in class | 0 |
| GeneSet | GOBP\_LEUKOCYTE\_HOMEOSTASIS |
| Enrichment Score (ES) | -0.5641687 |
| Normalized Enrichment Score (NES) | -1.887887 |
| Nominal p-value | 0.0 |
| FDR q-value | 0.07746566 |
| FWER p-Value | 0.492 |
Table: GSEA Results Summary

  

Fig 1: Enrichment plot: GOBP\_LEUKOCYTE\_HOMEOSTASIS      
 Profile of the Running ES Score & Positions of GeneSet Members on the Rank Ordered List

  

| SYMBOL | TITLE | RANK IN GENE LIST | RANK METRIC SCORE | RUNNING ES | CORE ENRICHMENT || 1 | ANXA1 | na | 267 | 0.970 | 0.0064 | No |
| 2 | BAK1 | na | 775 | 0.598 | -0.0183 | No |
| 3 | NKX2-3 | na | 818 | 0.584 | -0.0045 | No |
| 4 | MERTK | na | 1875 | 0.293 | -0.0842 | No |
| 5 | BCL2 | na | 2059 | 0.261 | -0.0918 | No |
| 6 | TNFSF13B | na | 2307 | 0.222 | -0.1059 | No |
| 7 | PDE4B | na | 3069 | 0.117 | -0.1660 | No |
| 8 | DNAJA3 | na | 3136 | 0.109 | -0.1683 | No |
| 9 | ADAM17 | na | 3490 | 0.064 | -0.1960 | No |
| 10 | PPP2R3C | na | 3558 | 0.058 | -0.1999 | No |
| 11 | PMAIP1 | na | 3864 | 0.020 | -0.2248 | No |
| 12 | TCIRG1 | na | 3866 | 0.020 | -0.2243 | No |
| 13 | SLC15A4 | na | 4085 | -0.004 | -0.2424 | No |
| 14 | JAM3 | na | 4165 | -0.013 | -0.2486 | No |
| 15 | TNFRSF13B | na | 4829 | -0.084 | -0.3016 | No |
| 16 | HIF1A | na | 5064 | -0.109 | -0.3179 | No |
| 17 | BAP1 | na | 5243 | -0.125 | -0.3291 | No |
| 18 | BCL10 | na | 6214 | -0.226 | -0.4035 | No |
| 19 | CASP3 | na | 6448 | -0.248 | -0.4157 | No |
| 20 | JAK3 | na | 6534 | -0.258 | -0.4152 | No |
| 21 | ABL1 | na | 6549 | -0.259 | -0.4087 | No |
| 22 | LMO1 | na | 6928 | -0.300 | -0.4314 | No |
| 23 | ZC3H8 | na | 7308 | -0.342 | -0.4530 | No |
| 24 | HMGB1 | na | 7363 | -0.348 | -0.4472 | No |
| 25 | RPS6 | na | 7644 | -0.376 | -0.4595 | No |
| 26 | BCL2L11 | na | 8046 | -0.428 | -0.4803 | No |
| 27 | AKT1 | na | 8399 | -0.472 | -0.4958 | No |
| 28 | SOS1 | na | 8869 | -0.533 | -0.5192 | No |
| 29 | PPP3CB | na | 8981 | -0.547 | -0.5123 | No |
| 30 | MEF2C | na | 9538 | -0.637 | -0.5400 | No |
| 31 | GPAM | na | 9650 | -0.654 | -0.5299 | No |
| 32 | RC3H1 | na | 10061 | -0.721 | -0.5428 | Yes |
| 33 | AXL | na | 10109 | -0.730 | -0.5251 | Yes |
| 34 | SLC46A2 | na | 10160 | -0.743 | -0.5073 | Yes |
| 35 | PIK3CB | na | 10335 | -0.777 | -0.4988 | Yes |
| 36 | SKIL | na | 10339 | -0.778 | -0.4760 | Yes |
| 37 | NCKAP1L | na | 10411 | -0.792 | -0.4585 | Yes |
| 38 | TNFAIP3 | na | 10555 | -0.821 | -0.4462 | Yes |
| 39 | PIK3CD | na | 10707 | -0.855 | -0.4334 | Yes |
| 40 | IL2RA | na | 10774 | -0.872 | -0.4131 | Yes |
| 41 | GPR174 | na | 10813 | -0.881 | -0.3902 | Yes |
| 42 | CD74 | na | 10874 | -0.901 | -0.3686 | Yes |
| 43 | FADD | na | 10928 | -0.915 | -0.3459 | Yes |
| 44 | GPR183 | na | 10984 | -0.933 | -0.3228 | Yes |
| 45 | KITLG | na | 10999 | -0.936 | -0.2963 | Yes |
| 46 | RC3H2 | na | 11093 | -0.963 | -0.2756 | Yes |
| 47 | MPL | na | 11201 | -1.000 | -0.2549 | Yes |
| 48 | ADA | na | 11226 | -1.008 | -0.2270 | Yes |
| 49 | LYN | na | 11314 | -1.043 | -0.2034 | Yes |
| 50 | SOS2 | na | 11362 | -1.063 | -0.1758 | Yes |
| 51 | RIPK3 | na | 11401 | -1.080 | -0.1470 | Yes |
| 52 | DOCK10 | na | 11592 | -1.169 | -0.1283 | Yes |
| 53 | SH2B3 | na | 11614 | -1.181 | -0.0950 | Yes |
| 54 | DOCK11 | na | 11721 | -1.262 | -0.0665 | Yes |
| 55 | FAS | na | 11850 | -1.412 | -0.0354 | Yes |
| 56 | FLT3 | na | 11939 | -1.644 | 0.0059 | Yes |
Table: GSEA details [plain text format]

  

Fig 2: GOBP\_LEUKOCYTE\_HOMEOSTASIS      
 Blue-Pink O' Gram in the Space of the Analyzed GeneSet

  

Fig 3: GOBP\_LEUKOCYTE\_HOMEOSTASIS: Random ES distribution      
 Gene set null distribution of ES for **GOBP\_LEUKOCYTE\_HOMEOSTASIS**
